# Respiratory distress syndrome and bronchopulmonary dysplasia after fetal growth restriction: Lessons from a natural experiment in identical twins

**DOI:** 10.1016/j.eclinm.2021.100725

**Published:** 2021-01-29

**Authors:** Sophie G. Groene, Jip A. Spekman, Arjan B. te Pas, Bastiaan T. Heijmans, Monique C. Haak, Jeanine M.M. van Klink, Arno A.W. Roest, Enrico Lopriore

**Affiliations:** a Neonatology, Dept. of Pediatrics, Leiden University Medical Center, Leiden, the Netherlands; b Molecular Epidemiology, Dept. of Biomedical Data Sciences, Leiden University Medical Center, Leiden,, the Netherlands; c Fetal Therapy, Dept. of Obstetrics, Leiden University Medical Center, Leiden, the Netherlands; d Pediatric Cardiology, Dept. of Pediatrics, Leiden University Medical Center, Leiden, the Netherlands

**Keywords:** Selective fetal growth restriction, Respiratory distress syndrome, Bronchopulmonary dysplasia

## Abstract

**Background:**

Fetal growth restriction (FGR) is thought to negatively affect lung development resulting in increased respiratory morbidity. However, research performed in singletons is often limited by a certain level of bias caused by individual differences in genetic constitution, obstetrical and maternal factors.

**Methods:**

Respiratory morbidity was compared between the smaller and the larger twin in monochorionic twins with selective fetal growth restriction (sFGR), defined as a birth weight discordance ≥ 20%, born in our center between 2010 and 2019 in this retrospective study. Respiratory distress syndrome (RDS) was diagnosed based on the clinical picture of a neonate with respiratory failure requiring mechanical ventilation and/or surfactant, confirmed by a chest X-ray. Bronchopulmonary dysplasia (BPD) was diagnosed when the neonate required treatment with >21% oxygen for at least 28 days.

**Findings:**

Median gestational age at birth for the 94 included pregnancies was 32.4 (IQR 30.4–34.3) weeks. Within-pair analyses showed that the prevalence of RDS was lower in the smaller twin compared to the larger twin, 19.1% (18/94) vs 34.0% (32/94), respectively (*p* = 0.004). The odds of RDS for the larger twin was doubled (OR 2.1 (CI95% 1.3–3.5). In contrast, the rate of BPD in the smaller twin was higher as opposed to the larger twin, 16.7% (15/90) vs 6.7% (6/89), respectively (*p* = 0.008), with a more than doubled odds (OR 2.5 (CI95% 1.3–4.9)).

**Interpretation:**

Despite being genetically identical, sFGR twins have different respiratory outcomes. Adverse growth condition *in utero* in the smaller twin is associated with a reduced odds of RDS at birth but a more than doubled odds of BPD, reflecting the pathophysiologic adverse effect of growth restriction on lung development.

**Funding:**

The Dutch Heart Foundation (2017T075).

**Research in context**

**Evidence before this study**

Evidence suggests that chronic intrauterine stress, as caused by fetal growth restriction (FGR), influences lung development throughout pregnancy and thereby possibly lead to lifelong changes in respiratory functioning. Animal models have shown that FGR can induce persistent changes to both the lung and chest wall, already impairing respiratory function in early postnatal life. Previous research in singletons has identified FGR as a risk factor for both progressive respiratory insufficiency directly after birth, and bronchopulmonary dysplasia.

**Added value of this study**

This study evaluated respiratory morbidity in a unique population of monochorionic twins with selective fetal growth restriction (sFGR). Despite being genetically identical, sFGR twins have different respiratory outcomes. Adverse growth condition *in utero* in the smaller twin is associated with a reduced risk of respiratory distress at birth but a more than doubled risk of bronchopulmonary dysplasia (BPD).

**Implications of all the available evidence**

These insights in a unique population of identical twins, press the pathophysiologic adverse effect of growth restriction on lung development.

Alt-text: Unlabelled box

## 1 Introduction

Chronic intrauterine stress, as caused by fetal growth restriction (FGR), is thought to influence lung development and thereby lead to lifelong changes in respiratory functioning. Animal models have shown that FGR can induce persistent changes to both the lung and chest wall, already impairing respiratory function in early postnatal life [1]. Moreover, previous research in singletons has identified FGR as a risk factor for both respiratory distress syndrome (RDS), characterized by progressive respiratory insufficiency directly after birth, and bronchopulmonary dysplasia (BPD) which is in turn associated with chronic lung disease in adulthood [2], [3], [4]. In contrast, it is also hypothesized that FGR is protective for RDS by way of an increased endogenous surfactant production resulting in enhanced lung maturation [5].

Although many studies have tried to uncover the effect of FGR on respiratory morbidity, results are often contradictory as a multitude of other factors can influence respiratory functioning beyond FGR. Research performed in singletons is therefore often limited by a certain level of bias caused by individual differences in genetic constitution, obstetrical and maternal factors (maternal diseases and medication). Hence, conclusive evidence is still lacking. A unique population in which these potential confounders are naturally eliminated are monochorionic (MC) twins.

**Fig. 1:**
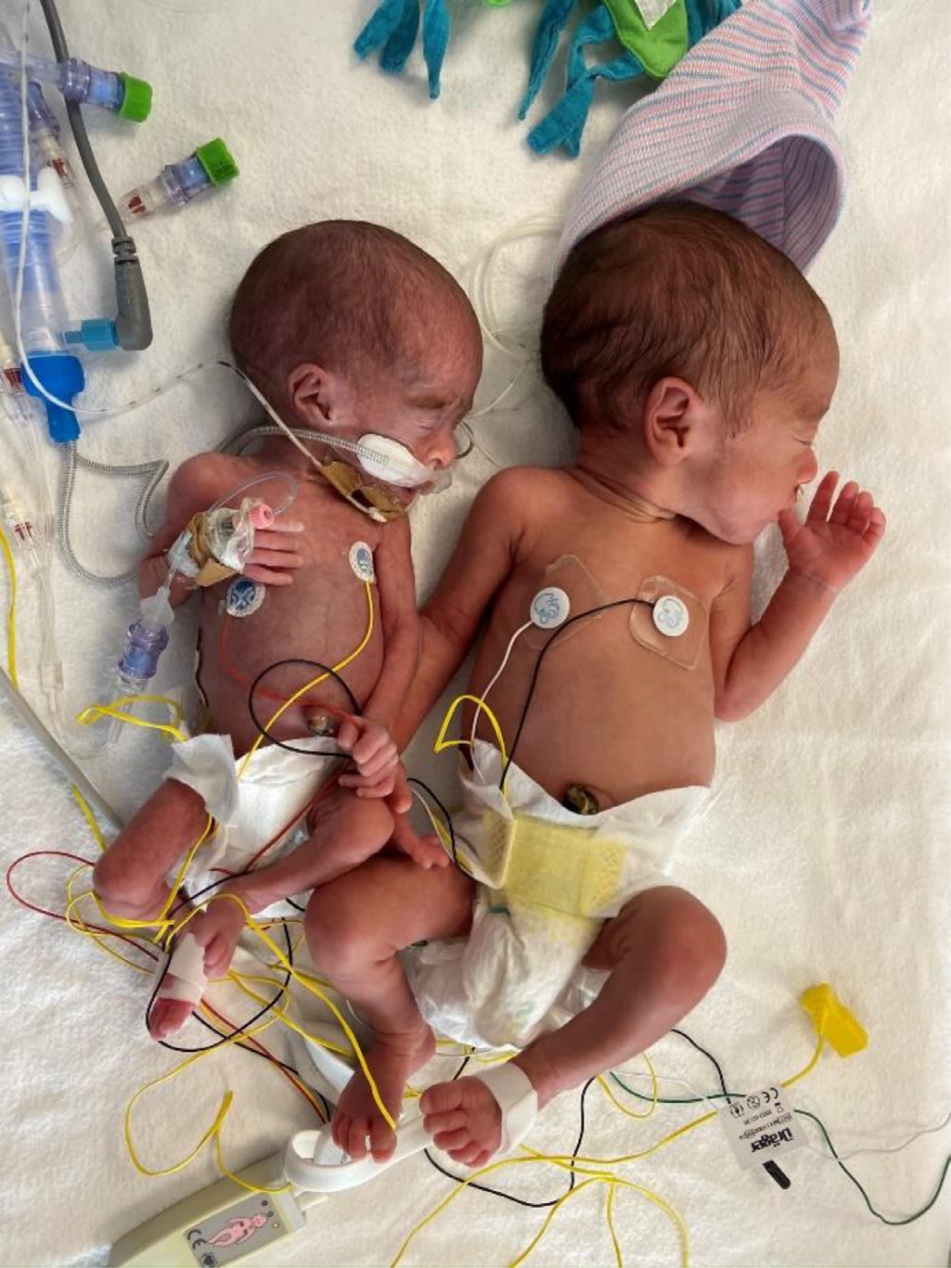
A MC twin pair with sFGR born at a gestational age of 30 weeks with respective birth weights of 500 gs (left) and 1350 gs (right). The picture was taken ten days after birth.

MC twins are vulnerable to perinatal complications because of the shared placenta with vascular anastomoses [6]. In 10–15% of MC twin pregnancies, the placenta is unequally shared leading to a large intertwin growth discrepancy described as selective fetal growth restriction (sFGR) (Fig. 1) [7,8]. As MC twins are genetically identical and also have identical maternal factors, they present the ideal natural experiment to study the effect of adverse “environmental” intrauterine circumstances. So far, only few small studies have thoroughly explored the respiratory outcomes in MC twins with sFGR [9]. In this study we aim to compare respiratory outcomes, primarily RDS and BPD in which we expect to find consistent associations, between the larger and smaller twin in a large cohort of MC twins with sFGR.

## 2 Methods

This report is written according to the STROBE guidelines [10]. The study was approved by the ethics committee of the Leiden University Medical Center (LUMC) and waived of the requirement for written informed consent due to its retrospective design. All consecutive live-born MC twin pairs born in the LUMC between 2010 and 2019 were eligible for inclusion. The LUMC is the national referral center for complicated MC twin pregnancies and fetal therapy in the Netherlands. The cases were reviewed for the presence of sFGR, defined as a birth weight discordance (BWD) ≥ 20%. BWD was calculated as: (birth weight larger twin – birth weight smaller twin)/birth weight larger twin X 100% [11]. All MC twins with sFGR were included. We excluded MC triplet pregnancies, cases with twin reversed arterial perfusion (TRAP) [12] and cases with other congenital abnormalities including, but not limited to heart defects, neural tube defects and chromosome abnormalities. Cases with twin anemia-polycythemia sequence (TAPS) [13], twin-twin transfusion (TTTS) cases who underwent treatment other than laser coagulation (no treatment, amniocentesis, and/or selective reduction) and cases with incomplete laser (either recurrent TTTS or post-laser TAPS) were excluded as well due to their added adverse intrauterine circumstances.

The following maternal and neonatal baseline characteristics were collected from medical records: maternal age, gravidity, parity, presence of underlying maternal health problems, occurrence of preeclampsia (defined as new-onset hypertension in the setting of proteinuria during pregnancy) [14], occurrence of gestational diabetes (defined as any degree of glucose intolerance with onset during pregnancy) [15], suspected chorioamnionitis (based on the following clinical criteria: maternal intrapartum fever, maternal leukocytosis, purulent cervical drainage, or fetal tachycardia) [16], presence of umbilical artery (UA) Doppler abnormalities (defined as persistent or intermittent absent or reversed end-diastolic flow (A/REDF)) [17], occurrence of TTTS (diagnosed according to the Eurofoetus criteria [18]), gestational age at diagnosis of TTTS, gestational age at laser coagulation, amnionicity, gestational at birth, sex, delivery mode, presence of fetal distress at birth (defined as abnormal cardiotocography (CTG)), whether a full course of corticosteroids was administered prior to delivery and BWD. Birth weight and the proportion of neonates that were born small for gestational age (SGA) (defined as birth weight < 10^th^ centile) were compared between the larger and the smaller twin. Differences in baseline characteristics for cases with and without TTTS were evaluated as well.

All MC twin placentas were routinely injected with colored dye as part of standard care to evaluate vascular patterns and placental share [19]. Fetal territories were distinguished by either the laser demarcation line, or the margins of the twin-specific dyes and expressed as a percentage of the total placental area measured using Image J version 1.57.

Primary outcomes were the prevalence of RDS and BPD. RDS was diagnosed based on the clinical picture of a neonate with respiratory failure receiving mechanical ventilation and/or surfactant, confirmed by a chest X-ray [20]. BPD was diagnosed when the neonate required treatment with > 21% oxygen for at least 28 days [21].

Secondary outcomes included persistent pulmonary hypertension of the newborn (PPHN) (defined as the failure of circulatory transition after birth requiring treatment with nitric-oxide (NO) [22]), patent ductus arteriosus (PDA) requiring medical treatment or surgical closure, necrotizing enterocolitis (NEC) ≥ stage 2 [23], neonatal sepsis (defined as a clinically ill neonate with positive blood cultures), asphyxia [24] (defined as at least three of the following criteria: signs of fetal distress before delivery; Apgar score 〈 5 at 5 min; arterial pH < 7.1 and base excess ≥ 16 mmol/L or lactate 〉 10 mmol/L in either arterial umbilical cord blood or capillary blood gas sample within 1 h after birth; respiratory failure requiring resuscitation measures during at least 5 min after birth; multiple organ failure), neonatal mortality (defined as mortality within the first 28 days after birth), Apgar scores at 1, 5 and 10 min, proportion of twins requiring major resuscitation at birth (defined as chest compression and/or epinephrine administration) proportion of twins intubated at birth, proportion of twins with continuous positive airway pressure (CPAP) during transport to the neonatal intensive care unit (NICU), proportion of twins who received surfactant, whether surfactant was administrated via minimally invasive surfactant therapy (MIST), number of doses of surfactant, proportion of twins who received mechanical ventilation, duration of mechanical ventilation, day of start after birth of mechanical ventilation, proportion of twin who received high frequency oscillation (HFO), duration of HFO, proportion of twins who received CPAP, duration of CPAP, proportion of twins who received non-invasive respiratory support (defined as CPAP, high flow and low flow), duration of non-invasive respiratory support, the total duration of respiratory support until discharge to home (including both non-invasive respiratory support and mechanical ventilation), duration of NICU admission and respiratory support at discharge from the NICU (either no respiratory support or non-invasive respiratory support). The outcomes were compared between the larger and the smaller twin within each twin pair.

Statistical analyses were performed using IBM SPSS Statistics Version 25.0 (SPSS, Inc., an IBM company, Chicago, IL, USA). Data are presented as median (interquartile range), n/N (%) or n (%). To test for association between FGR and neonatal morbidity and mortality (categorical data), we used a Generalized Estimated Equation (GEE) to analyze within-pair differences. The association between FGR and type of respiratory support was analyzed similarly. When an outcome was not observed in one of the groups, an adjustment to the data was applied in which an unaffected twin was changed into an affected twin for both groups, as the GEE cannot be used when an outcome event does not occur in one of the groups. This adjustment generates more conservative *p*-values. The analysis of RDS was corrected for birth order, as it has previously been described that the firstborn in twin pregnancies is more susceptible to RDS [25,26]. To test for association between FGR and start/duration of respiratory support (numerical data), a Wilcoxon Signed Rank Test was used to assess within-pair differences. Both the GEE and the Wilcoxon Signed Rank Test account for the fact that observations between co-twins are not independent. We did not correct for multiple testing, because our primary outcomes (BPD and RDS) share etiological factors and are thereby not independent but related. So, we expect to observe consistent associations between our outcomes of interest. Differences in baseline characteristics between cases with and without TTTS were analyzed using a Mann-Whitney-U (numerical data) or a Chi-square test (categorical data). A *p*-value of < 0.05 was considered statistically significant.

### 2.1 Role of funding

This research was funded by The Dutch Heart Foundation, grant number 2017T075. The funding source had no role in study design; in the collection, analysis, and the interpretation of the data; in the writing of the report; and in decision to submit the paper for publication.

## 3 Results

**Fig. 2:**
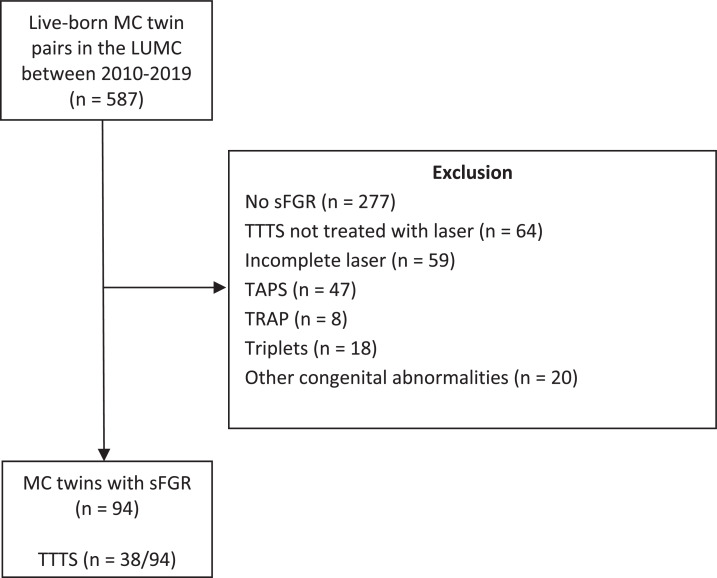
Flowchart of study inclusion.

A total of 587 live-born MC twin pairs were delivered at the LUMC between 2010 and 2019 (Fig. 2). After exclusion according to the aforementioned criteria (*n* = 216) and the cases without sFGR (*n* = 277), 94 twin pairs with sFGR were included for analysis.

Maternal and neonatal baseline characteristics are summarized in Table 1. Preeclampsia was diagnosed in 7.4% (7/94) and gestational diabetes in 5.3% (5/94) of pregnancies. Chorioamnionitis was suspected in 8.5% (8/94) of pregnancies. UA Doppler abnormalities were documented in 50 pregnancies, of which 20.2% (19/94) were persistent A/REDF and 33.0% (31/94) were intermittent A/REDF. Nearly half (40.4% (38/94)) of the included twin pairs were diagnosed with TTTS and successfully treated with laser therapy. Median gestational age at birth was 32.3 (30.4–34.3) weeks, and 45.7% (43/94) was born < 32 weeks of gestation. The twins were delivered by way of cesarian section in 66.0% (124/188) of cases. Fetal distress was present in 50% (47/94) of cases and 72% (67/93) received a full course of corticosteroids prior to delivery. The median BWD was 31.8% (26.7–40.8). The median birth weight of the large twin was 1819 (1464–2336) and 1183 (912–1490) grams for the smaller twin. In the group of smaller twins, the proportion of neonates born SGA was 97.9% (92/94) as opposed to 13.8% (13/94) in the group of larger twins. The larger twin had a significantly larger placental share (68.9% (63.0–73.9)) compared to the smaller twin (31.1% (26.1–37.0)).

**Table 1:** Baseline maternal and neonatal characteristics for sFGR twins.

Characteristics	sFGR twins (*n* = 188; 94 pregnancies)	Larger twin (*n* = 94)	Smaller twin (*n* = 94)
Maternal age – *years*	31 (28–35)		
Gravidity	2 (1–3)		
Parity	0 (0–1)		
Maternal health problems	7/94 (7.4)		
Pregnancy complications			
Preeclampsia	7/94 (7.4)		
Gestational diabetes	5/94 (5.3)		
Suspected chorioamnionitis	8/94 (8.5)		
UA Doppler abnormalities			
A/REDF	19/94 (20.2)		
iA/REDF	31/94 (33.0)		
TTTS	38/94 (40.4)		
Gestational age at diagnosis TTTS – *weeks*	18.5 (16.4–20.2)		
Gestational age at laser – *weeks*	18.7 (16.7–20.8)		
Monoamniotic twins	3/94 (3.2)		
Gestational age at birth – *weeks*	32.3 (30.4–34.3)		
Gestational age at birth < 32 weeks	43/94 (45.7)		
female	88/188 (46.8)		
cesarian	124/188 (66.0)		
Fetal distress	47/94 (50.0)		
Full course of corticosteroids prior to delivery	67/93 (72.0)		
Birth weight discordance –*%*	31.8 (26.7–40.8)		
Birth weight – *grams*		1819 (1464–2336)	1183 (912–1490)
Small for gestational age		13/94 (13.8)	92/94 (97.9)
Placental share –*%*		68.9 (63.0–73.9)	31.1 (26.1–37.0)

sFGR: selective fetal growth restriction, UA: umbilical artery, A/REDF: absent or reversed end-diastolic flow, iA/REDF: intermittent absent or reversed end-diastolic flow, TTTS: Twin-twin transfusion syndrome. Outcomes are presented as median (interquartile range (IQR)) or n/N (%).

Neonatal morbidity and respiratory outcomes for the larger and smaller twin are presented in Table 2 and 3, respectively. The Apgar score at one minute was 7 (5–8) for the small twin and 8 (6–9) for the large twin (*p* < 0.0001). The prevalence of RDS was lower in the smaller twin compared to the larger twin (19.1% (18/94) vs. 34.0% (32/94), *p* = 0.004). The odds of RDS for the larger twin were doubled (OR 2.1 (CI95% 1.3–3.5). The large twin required surfactant more often, in 27.7% (26/94) of cases, compared to 10.8% (10/93) of cases in the smaller twin (*p* < 0.0001). The median duration of mechanical ventilation for the smaller twin was 8 (1–18) days as opposed to 2 (1–4) days for the larger twin (*p* = 0.016). The median duration of NICU admission was 6 (2–14) days for the smaller twin and 7 (3–20) days for the larger twin (*p* = 0.001).

**Table 2:** Neonatal morbidity in the larger versus the smaller twin.

Outcomes	Larger twin (*n* = 94)	Smaller twin (*n* = 94)	p-Value
RDS	32/94 (34.0)	18/94 (19.1)	0.004*
BPD	6/89 (6.7)	15/90 (16.7)	0.008
PPHN	6/92 (6.5)	5/93 (5.4)	0.749
PDA	3/94 (3.2)	7/94 (7.4)	0.167
NEC	0/94 (0.0)	2/94 (2.1)	0.175
Sepsis	11/92 (12.0)	15/91 (16.5)	0.333
Asphyxia	1/94 (1.1)	2/94 (2.1)	0.571
Neonatal mortality	2/94 (2.1)	2/94 (2.1)	1.000

RDS: respiratory distress syndrome, BPD: bronchopulmonary dysplasia, PPHN: persistent pulmonary hypertension of the newborn, PDA: patent ductus arteriorus, NEC: necrotizing enterocolitis, CPAP: continuous positive airway pressure. Outcomes are presented as n/N (%).

*corrected for birth order.

**Table 3:** Respiratory outcomes in the larger versus the smaller twin.

Outcomes	Larger twin (*n* = 94)	Smaller twin (*n* = 94)	p-Value
Apgar			
1 min	8 (6–9)	7 (5–8)	<0.0001
5 min	9 (8–9)	9 (8–9)	0.282
10 min	9 (8–10)	9 (8–10)	0.816
Major delivery room resuscitation	0/94 (0.0)	1/93 (1.1)	0.324
Intubation at birth	7/92 (7.6)	7/91 (7.7)	0.985
CPAP to NICU	49/92 (53.3)	51/91 (56.0)	0.667
Surfactant	26/94 (27.7)	10/93 (10.8)	<0.0001
MIST	12/26 (46.2)	4/10 (40.0)	0.341
Number of doses	1 (1–2)	1 (1–2)	1.000
Mechanical ventilation	23/94 (24.5)	19/93 (20.4)	0.388
Day of start	1 (1–2)	1 (1–3)	0.750
Duration – *days*	2 (1–4)	8 (1–18)	0.024
HFO	4/94 (4.3)	7/93 (7.5)	0.314
Duration – *days*	2 (1–4)	7 (4–12)	*
CPAP	57/93 (57.3)	57/91 (51.6)	0.059
Duration – *days*	2 (1–5)	3 (1–11)	0.392
Non-invasive respiratory support	60/94 (63.8)	54/93 (58.1)	0.235
Duration – *days*	4 (1–10)	6 (1–31)	0.076
Total duration of respiratory support – *days*	5 (1–11)	4 (1–32)	0.110
Duration NICU admission	7 (3–20)	6 (2–14)	0.001
Respiratory support at discharge NICU			
No support	79/90 (87.8)	73/91 (80.2)	0.077
Non-invasive respiratory support	11/90 (12.2)	18/91 (19.8)	0.139

NICU: neonatal intensive care unit, MIST: minimally invasive surfactant therapy, HFO: high frequency oscillation. Outcomes are presented as median (IQR) or n/N (%).

*No within-pair comparison possible, due to low number of cases.

The prevalence of BPD was significantly higher in the smaller twin as opposed to the larger twin (16.7% (15/90) vs 6.7% (6/89), (*p* = 0.008). The odds of developing BPD for the smaller twin was more than doubled (OR 2.5 (CI95% 1.3–4.9)). The median gestational age at birth of the group with BPD was 28.6 (IQR 28.0–29.8). Of the large twins that developed BPD, 66.7% (4/6) received mechanical ventilation, as opposed to 46.7% (7/15) of the small twins. The median duration of the mechanical ventilation for the large twins with BPD was 3 (2–7) days vs. 14 (2–27) days for the small twins with BPD.

Differences in baseline characteristics were examined for cases with and without TTTS. Significant differences were found for the presence of fetal distress (34.2% (13/38) in cases with TTTS vs. 60.7% (34/56) in cases without TTTS, *p* = 0.012) and delivery mode (cesarian section in 50.0% (38/76) of cases with TTTS as opposed to 76.8% (86/112) in cases without TTTS, *p* < 0.0001). Furthermore, the presence of TTTS was associated with three outcomes, namely CPAP during transfer to the NICU (40.3% in TTTS cases as opposed to 64.5% (*p* = 0.004), still no difference between large and small twin), the duration of mechanical ventilation (no differences between large (2 (1–6) days) and small (3 (1–12) days) twin in TTTS cases (*p* = 0.285)) and the duration of NICU admission (no differences between large (6 (2–12) days) and small (7 (3–17) days) twin in TTTS cases (*p* = 0.326)).

## 4 Discussion

This study shows that while fetuses suffering from FGR may be ‘protected’ from acute respiratory morbidity at birth their odds of developing chronic respiratory morbidity is more than doubled. Our results originate from a simple but unique natural experiment in identical twins, comparing the respiratory outcomes in the smaller growth restricted twin to the larger co-twin. Despite their identical genetic constitution and identical maternal factors, their odds of specific respiratory morbidity differ significantly. We are the first to describe detailed respiratory outcomes, including BPD, in a cohort of monochorionic twins. Our data emphasize that an adverse intrauterine environment may have a pathophysiologic effect on neonatal lung development.

Multiple pathways have been implicated to explain the mechanism behind the respiratory pathology associated with FGR. A widely proposed hypothesis is that the smaller twin has an increased corticosteroid production as a result of prolonged exposure to prenatal stress, by which the smaller twin is better prepared for the neonatal transition following elective premature delivery as opposed to the large twin [27]. This is reflected in a lower prevalence of RDS for the smaller twin in our study population. One can speculate that the corticosteroids produced by the smaller twin can be transferred to the large twin through the vascular anastomoses [28]. When separately examining the lasered TTTS cases in our population, the significant difference in RDS rate for the larger and smaller twin is still present (13.5% in the larger twin vs. 28.8% in the smaller twin, *p* = 0.011), indicating a negligible effect of this transfer.

Simultaneously, FGR is thought to already negatively affect lung development *in utero* by inducing persistent structural and functional changes to the respiratory system associated with the pathophysiology of BPD [5,29]. Firstly, chronic hypoxia as caused by FGR, can impair pulmonary angiogenesis by way of reactive oxygen species and a down- or upregulation of key proteins (such as elastin and collagen, respectively) and growth factors (such as vascular endothelial growth factor) [30]. Pulmonary vascular resistance increases, in turn causing further cardiovascular adaptations amongst which increased vascular stiffness and cardiac hypertrophy. Secondly, the impaired vascularization disrupts the alveolarization as well, resulting in poor alveolar morphology [31]. Combined, gas exchange becomes inefficient [32,33]. So, the abnormal lung development associated with FGR increases the vulnerability for postnatal insults and thereby the risk of BPD.

A factor possibly complicating the interpretation of our data is the prolonged mechanical ventilation in the smaller twin, which has been identified as an independent risk factor for BPD [34]. One may speculate that the increased BPD rate for the smaller twin is not solely attributable to the FGR but also to the extensive mechanical ventilation following the presence of, as is largely the case in our population, severe neonatal morbidity. Nonetheless, animal models have shown that FGR does not further exacerbate ventilation induced lung injury [35]. Moreover, it is important to realize that both the development of complications and thereby the need for prolonged mechanical ventilation are closely interwoven with FGR as well. In addition, caretakers might be more prone to prolong ventilation based on the perceived vulnerability of SGA neonates, regardless of the true necessity for respiratory support at that point. As the etiology of BPD is multifactorial, similarly to the etiology of FGR, it is difficult to discern through which mechanisms the association between FGR and BPD truly runs. Still, there is an apparent association that should be taken into account in clinical practice.

Furthermore, the distinctive etiologies of FGR in singletons and sFGR in MC twins should be considered. While sFGR in MC twins is characterized by unequal placental sharing and thereby a small placental volume for the smaller twin, FGR in singletons comes from placental insufficiency caused by abnormal placentation. Impaired trophoblast invasion results in inadequate remodeling of the uterine spiral arteries, reducing uteroplacental blood flow [36]. So, the mechanisms of chronic hypoxia in FGR and sFGR differ significantly, possibly affecting outcomes.

Regarding twin research, our results are in agreement with previous studies identifying the presence of sFGR and being the larger twin as important risk factors for RDS [[25], [26], [27],^37^]. However, these studies all lack a distinction in chorionicity (and zygosity) and are thereby still limited by genetic constitution as a potential confounder. Moreover, information on the prevalence of BPD is often missing from these studies as well as a within-pair comparison. The only study on respiratory morbidity in growth discordant MC twins was performed at our center and found that the larger twin is at increased risk of RDS (32% vs. 6%), compared to the smaller twin [9]. Our results are complementary, albeit in a larger study population with a more thorough examination of additional respiratory outcomes including BPD. Another small study that conducted a within-pair comparison of twins with discordant fetal growth patterns (once more lacking a distinction in chorionicity and zygosity) has shown that the small twin had a significantly decreased lung function and an increased bronchial reactivity at the age of 16 years [38]. These results affirm the long-lasting effects of FGR that need to be researched more thoroughly.

The retrospective nature of our study should be taken into account when interpreting the results, as the documentation of BPD was largely based on hospital discharge letters. A timed oxygen reduction test which is the current golden standard in the diagnosis of BPD was only performed in 22% of BPD cases in our population [39]. Additionally, as the LUMC is the national referral center for complicated MC twin pregnancies referral bias may enlarge the proportion of twins with an anticipated severe neonatal course. On the other hand, our population is inherently at low risk of BPD due to the low number of extremely preterm neonates (gestational age at birth < 28 weeks and/or birth weight < 1000 gs), leading to relatively few neonates with BPD. This possibly limits the applicability of our results to higher risk preterm neonates and thereby also the use of newer BPD definitions [40]. Prospective research evaluating both neonatal respiratory morbidity and childhood lung function, not solely focusing on BPD cases as those without BPD are not necessarily free of respiratory morbidity [41], is necessary to provide more conclusive evidence. Nevertheless, the results of our study are strengthened by the extensive documentation of respiratory data and the size and unique nature of our study population, consisting of monozygotic twins discordant for intrauterine exposures that can thereby act as each other's control for genetic, maternal and obstetrical factors. Moreover, all twins were born in a single specialized center, limiting treatment variation and resulting in availability of information on resuscitation, surfactant supplementation and initial respiratory support.

In conclusion, in MC twins FGR is associated with a reduced risk of respiratory distress at birth but an increased the risk of BPD in the smaller twin, emphasizing the early consequences of FGR for lung structure and functioning. Our study helps improve the perhaps counterintuitive anticipation of respiratory problems after birth for both pediatricians and parents. It also provides a basis for risk assessment of chronic lung disease in childhood and throughout adulthood, pressing the need for larger, prospective studies on respiratory morbidity in MC twins with sFGR to further research the early origin of lung disease.

## Declaration of Competing Interest

No conflicts of interest.

## Appendix Supplementary materials

Image, application 1
